# Reply to Ye: Parallel progress in funder practices and expansion of academic incentives to support engaged research

**DOI:** 10.1073/pnas.2500539122

**Published:** 2025-02-27

**Authors:** Angela T. Bednarek, Ben Miyamoto

**Affiliations:** ^a^Scientific Advancement, The Pew Charitable Trusts, Washington, DC 20004

We appreciate Ye’s comments and the opportunity they provide to share more about the Transforming Evidence Funders Network’s (TEFN) efforts related to research incentives and reward systems (1). Our article focused on grant practices as an important lever for change (2). And while funder practices have a part to play in supporting engaged research, there are other changes needed, including the expansion of research incentives (3, 4).

Today, engaged research is not consistently valued in faculty reward systems that still primarily emphasize scholarly contributions and outputs. In addition, we have heard from funders in TEFN that these misaligned incentives posed a system-wide barrier to supporting more engaged research. Indeed, several TEFN funders already have longstanding grant programs that aim to expand incentives to enable and incentivize researchers to participate in engaged research, including the William T. Grant Foundation’s Institutional Challenge Grant program and the Carnegie Corporation of New York’s Bridging the Gap program (3).

Seeing potential to build on these efforts and spur broader change, TEFN commissioned a landscape scan in 2023 of promising changes in promotion and tenure systems in the United States to better support research such as engaged research (5). In parallel, TEFN convened other key leaders from across the research ecosystem (e.g., professional and disciplinary societies, academic publishers, and others) to identify barriers and opportunities for change (6).

These efforts identified that engagement from funders can help accelerate innovative institutional change work already being led by faculty, administrators, students, and other partners. To boost strategic coordination across sectors, TEFN launched the University Presidents and Chancellors’ Council on Public Impact Research. This new North American initiative, led by Penn State University’s president, Neeli Bendapudi, supports collaborations between funders and university leaders to create a roadmap for promoting and rewarding public impact research. The roadmap aims to build on promising progress in broadening promotion and tenure systems and strengthen training and engagement infrastructure (7, 8).

TEFN is also aggregating insights about promising approaches to incentivizing public impact research from around the world. For example, the International Development Research Centre is supporting a landscape scan of research reward systems in universities in Southern African countries (9). And TEFN is working to identify promising progress in other settings, including initiatives like the U.K. Research Excellence Framework 2029 and the efforts of the Global Research Council’s Responsible Research Assessment Working Group (10, 11).

We agree with Ye that reshaping research incentives is critical for supporting engaged research. And we know that the projects above are just a few examples of many that aim to strengthen research culture and incentives to support researchers’ public impact contributions. Even as TEFN participants look inward at their own funding practices, funders are also looking outward to collaborate with higher education, government, and other sectors to take full advantage of research’s potential to contribute to societal problem-solving. We are grateful for Ye’s letter and for the opportunity to expand on this essential topic.
